# Home and Neighborhood Context and Fall Risk Among Older Americans

**DOI:** 10.1093/geroni/igaa057.2503

**Published:** 2020-12-16

**Authors:** Safiyyah Okoye, John Mulcahy, Chanee Fabius, Jennifer Wolff

**Affiliations:** Johns Hopkins Bloomberg School of Public Health, Baltimore, Maryland, United States

## Abstract

Falls result from complex interactions between individuals and their environment and are the leading cause of injuries among older adults. A nascent literature demonstrates an association between neighborhood characteristics and falls. However, available evidence is from small, nonrepresentative samples and generally focuses on individual, home, or neighborhood risk-factors rather than the contribution of all three. We link information from N=6,489 community-dwelling participants in the 2015 National Health and Aging Trends Study with the Social Deprivation Index (SDI), which yields a census-tract-level score of socioeconomic disadvantage, to assess associations between home and neighborhood context and falls in the previous year. Household financial strain was associated with a 31% increased risk of falling, and indoor trip hazards with a 14% increased risk, after adjusting for individual factors and neighborhood SDI (all p <0.05). Findings reflect the interplay between home and neighborhood context and fall-risk, and can inform community-based fall-prevention interventions.

